# Roentgen Rays

**Published:** 1899-09-02

**Authors:** 


### ROENTGEN RAYS.

Speaking at the last meeting of the Roentgen Society, Dr. Mansell Moullin said that there was no branch of surgery which did not afford abundant evidence of the improvements which have taken place in the production and utilisation of the Roentgen rays in the course of the past year. The screen had now reached such a degree of perfection that with suitable apparatus the minutest movements of the heart and lungs, and the least change in the action of the diaphragm, could be watched and studied in the living subject. Photographs of the most deeply buried bones could now be obtained without difficulty. Measurements of such structures as the pelvis could be taken without subjecting the patient to the least inconvenience, and the clinical records of the past year were full of instances showing what could be done in medical and surgical practice. Aneurisms, interlobular empyemata, mediastinal abscesses, and patches of central pneumonia could now be shown upon the screen with absolute accuracy, and so steady was the illumination that the earliest stages of tuberculous lesions in the lungs could be seen and recognised. Cavities also in the lungs, whether containing air or pus, could now be detected at once, and their position and depth from the surface could be accurately marked out, and even the existence of cysts or tumours, projecting from the upper surface of the liver and raising the diaphragm, could be demonstrated with the greatest clearness. Owing to the use of the rays military surgery, he said, would have to be re-written, so great was their utility in the localising bullets and foreign bodies. In civil practice, also, foreign bodies of great variety, such as plates of false teeth, Murphy's buttons, splinters of bone, pins and needles of various kinds, wire sutures, fragments of glass which may perhaps have been buried for years, and numberless other substances have been discovered in the body, and have been located as accurately as if they had been lying in some perfectly transparent medium.

